# Realtime PCR Is More Sensitive than Multiplex PCR for Diagnosis and Serotyping in Children with Culture Negative Pneumococcal Invasive Disease

**DOI:** 10.1371/journal.pone.0009282

**Published:** 2010-02-19

**Authors:** Chiara Azzari, Maria Moriondo, Giuseppe Indolfi, Martina Cortimiglia, Clementina Canessa, Laura Becciolini, Francesca Lippi, Maurizio de Martino, Massimo Resti

**Affiliations:** Department of Pediatrics, Anna Meyer Children's Hospital and University of Florence, Florence, Italy; Columbia University, United States of America

## Abstract

**Background:**

Pneumococcal serotyping is usually performed by Quellung reaction, considered the gold standard test. However the method cannot be used on culture-negative samples. Molecular methods can be a useful alternative. The aim of the study was to evaluate the use of Multiplex-sequential-PCR (MS-PCR) or Realtime-PCR on blood samples for diagnosis and serotyping of invasive pneumococcal disease (IPD) in a pediatric clinical setting.

**Methodology/Principal Findings:**

Sensitivity and specificity of MS-PCR and Realtime-PCR have been evaluated both on 46 well characterized pneumococcal isolates and on 67 clinical samples from children with culture-negative IPD. No difference in sensitivity and specificity between MS-PCR and Realtime PCR was found when the methods were used on isolates: both methods could type 100% isolates and the results were always consistent with culture-based methods. On the contrary, when used on clinical samples 43/67 (64.2%) were typeable by MS-PCR and 61/67 (91.0%) by Realtime-PCR (p = 0.0004,K Cohen 0.3, McNemar's p<0.001). Non-typeability by MS-PCR was associated in 18/20 cases (90.0%) with low bacterial load. The difference between the two methods was present both when they were used on normally sterile fluids (respectively 31/33 (93.9%) typeable samples for Realtime-PCR and 24/33 (72.7%) for MS-PCR, p = 0.047, 95%CL 0.03–0.98; K Cohen 0.3; McNemar's p = 0.0016) and when they were used on nasopharyngeal swabs (respectively 30/34 (88.2%) typeable samples for Realtime-PCR and 19/34 (55.9%) for MS-PCR, p = 0.007, 95%CL 0.04–0.66); the presence of multiple pneumococcal serotypes in nasopharyngeal swabs was found more frequently by Realtime PCR (19/30; 63.3%) than by Multiplex-sequential PCR (3/19; 15.8%; p = 0.003;95%CL 1.87–39.97).

**Conclusions/Significance:**

In conclusion, both MS-PCR and Realtime PCR can be used for pneumococcal serotyping of most serotypes/serogroups directly on clinical samples from culture-negative patients but Realtime-PCR appears more sensitive.

## Introduction

*Streptococcus pneumoniae* is considered the leading cause of invasive bacterial infections both in children and in elderly people [1]. The organism causes at least 1.6 million deaths each year and is responsible for several invasive infections [2]. Capsular polysaccharide helps to differentiate *Streptococcus pneumoniae* into 92 distinct serotypes [3]. Effective vaccines induce specific anti-capsular antibodies [4]. The available vaccine currently used in pediatric age induces protection against 7 major serotypes and new vaccines against 10 or 13 serotypes will be available soon. Individuating serotypes causing invasive infections is extremely important in order to understand pneumococcal epidemiology, plan correct vaccination programs and evaluate the impact of the existing program. On the other hand, molecular typing is an important tool also in studies on pneumococcal carriage and especially in studies focusing on serotype replacement occurring spontaneously or as a consequence of immune pressure induced by vaccination.

Pneumococcal serotyping is currently performed by using capsular swelling (Quellung) reaction, considered the traditional ‘gold standard’ [5]–[6]. However, cross-reactions between serotypes can occur and some strains remain non-serotypable [7]–[9]. Technical expertise requirements as well as the need for viable bacteria may be drawbacks of the culture detection-system. Actually, autolysis in culture media and antibiotic therapy often performed on an outpatient basis at the beginning of febrile illness, prevent pathogens from growing in culture plates so making diagnosis and serotyping with culture methods more difficult. Molecular methods, based on DNA detection, do not require viable bacteria so that can be considered as useful help to cultural methods especially in cases when antimicrobial treatment was already started [10].

As for carriers, studies aimed to evaluate pneumococcal serotype replacement need to consider the presence of multiple serotypes in nasopharyngeal swabs. However individuation and typing of pneumococci in nasopharyngeal swabs is usually obtained by a single colony method [11] on culture plates. On that way, a single serotype per patient is usually found and multiple colonization, which is a very frequent condition, cannot be revealed [12]. Colonization studies in which several colonies are studied are much more informative, but much more expensive.

In recent years, molecular techniques have been applied successfully in the identification of infectious agents. The knowledge of DNA/RNA sequences of most bacteria allows to obtain etiologic diagnosis [13] and bacterial serotyping even where standard culture methods fail [10], [14]–[15].

We have previously described that multiplex sequential PCR (MS-PCR) performed directly on clinical samples (both blood and other normally sterile fluids) for pneumococcal serotyping has a sensitivity which is significantly higher than that of culture [14]. However, even though the possibility of pneumococcal serotyping has been greatly ameliorated by MS-PCR, it could be further increased by the use of Realtime-PCR known, at present, as one of the most specific and sensitive molecular method [16]–[18].

The aim of the present study was to compare sensitivity and specificity of Realtime-PCR versus MS-PCR for pneumococcal typing both on isolates and on clinical samples from patients with culture-negative invasive pneumococcal disease.

## Results

### Molecular diagnosis of *Streptococcus pneumoniae*

#### Isolates

All the pneumococcal isolates (either those obtained from ATCC or those obtained from the Microbiology Laboratory) resulted positive with both RT PCR (*lytA* gene) and MS-PCR (*CpsA* gene).

#### Biological samples

All the samples were positive for *lytA* gene. Positivity for both *lytA* in Realtime-PCR and *CpsA* in MS-PCR was found in 67/69 (97.1%) samples (4 CSF, 4 pleural fluids, 25/27 blood samples, 34 pharyngeal swabs). The two samples (blood) negative in MS-PCR had a Realtime-PCR C*_T_* respectively of 38 and 39. Only the 67 samples positive for both *lytA* gene in Realtime-PCR and *CpsA* gene in MS-PCR were included in the study for serotyping analysis.

### Serotyping of *Streptococcus pneumoniae* Isolates

All the eight isolates obtained from ATCC could be serotyped by both molecular methods and the results were consistent with ATCC serotype.

All the 38 *Streptococcus pneumoniae* isolates could be serotyped by both molecular methods and showed a complete concordance with serologic typing (serotypes 1, 3, 4, 5, 6A: 3 isolates/each; serotypes 6B,7F, 8, 9V, 12F, 14: 2 isolates/each; serotypes 10, 15B/C, 18C, 19A, 19F, 20, 22F, 23F, 33, 35B, 38: 1 isolate/each). No cross-reaction between serotypes was found for any primer/probe set.

### Serotyping of *Streptococcus pneumoniae* from Biological Samples

**Figure 1:**
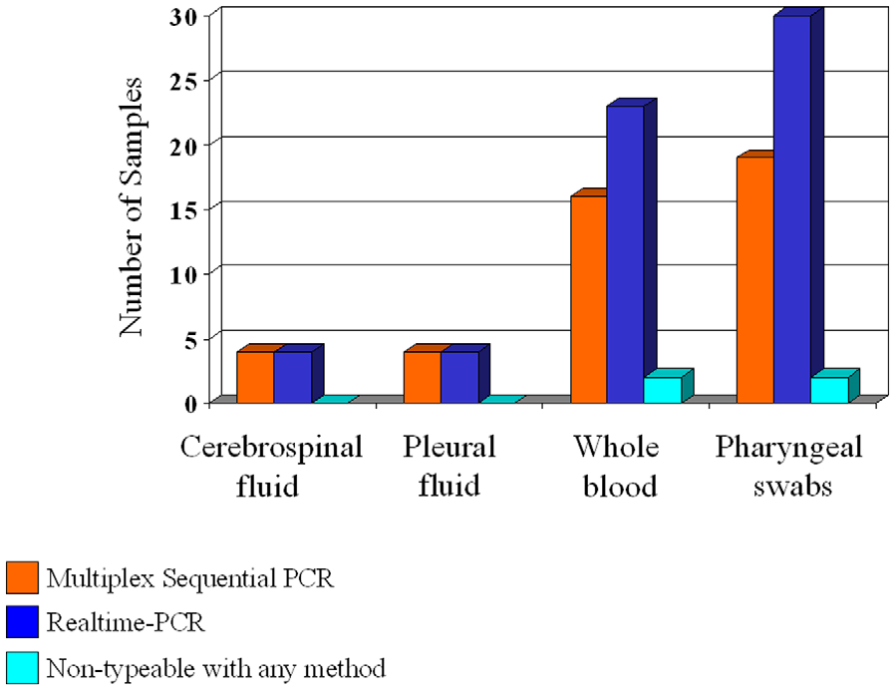
Number of typeable or non-typeable samples obtained from patients with culture-negative invasive pneumococcal infections as evidenced by Multiplex Sequential PCR or Realtime-PCR (sensitivity of Realtime vs Multiplex Sequential PCR p = 0.0004; 95%CL 1.98–17.05).

Among the 67 biological samples found positive for *Streptococcus pneumoniae*, 43/67 (64.2%) could be serotyped by MS-PCR, while 61/67 (91.0%) (p = 0.0004; 95%CL 0.06–0.51), could be serotyped by RT-PCR (K Cohen:0.3; McNemars's p<0.001). All CSF and pleural fluid samples could be serotyped with both molecular methods (Figure 1).

Among the 67 samples, 41 were typeable with both methods, 2 were typeable only by MS-PCR, 20 were typeable only by Realtime-PCR and 4 were not typeable with any method.

Among the 20 samples (7/25 whole blood, 13/34 pharyngeal swabs) which could be typed only with Realtime-PCR, 18/20 had a C*_T_* (threshold cycle) for *lytA* gene comprised between 30 and 38 (median 34.5; SD 2.4; with no difference between blood samples and swabs) so suggesting a low bacterial load, while 2 samples (pharyngeal swabs) had respectively a C*_T_* for *lytA* of 29 and 24.

The 2 samples (pharyngeal swabs) which could be serotyped only by MS-PCR were respectively serotypes 6C and 11A, primers of which were not available for RT-PCR at the time of the study.

A statistically significant difference between the two methods was present both when they were used on normally sterile fluids (blood, CSF, pleural fluid) or on nasopharyngeal swabs. When used on normally sterile fluids 24/33 (72.7%) samples were typeable with MS-PCR and 31/33 (93.9%)with Realtime-PCR (p = 0.047, 95%CL 0.03–0.98; K Cohen 0.3; McNemar's p = 0.0016); when the two methods were used on nasopharyngeal swabs 19/34 (55.9%) samples were typeable by MS-PCR and 30/34 (88.2%) by Realtime-PCR (p = 0.007, 95%CL 0.04–0.66).

**Figure 2:**
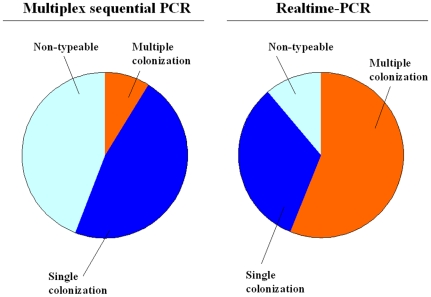
Presence of single or multiple pneumococcal serogroups/serotypes in nasopharyngeal swabs as evidenced by Multiplex Sequential PCR or Realtime-PCR (frequency of multiple serotypes/serogroup evaluated with Realtime PCR vs Multiple Sequential PCR p = 0.003; 95%CL = 1.87–39.97).

Multiple serotypes/serogroups of Streptococcus pneumoniae were demonstrated in 3/19 (15.8%) swabs typed by MS-PCR and in 19/30 (63.3%) pharyngeal swabs typed by Realtime-PCR (Figure 2), (p = 0.003; 95%CL = 1.87–39.97). MS-PCR demonstrated 2 different serotypes in 3/19 (15.8%), while in 16/19 samples (84.2%) only one serotype was found. Realtime PCR demonstrated 1 single serotype in 11/30 (36.7%), 2 different serotypes in 13/30 (43.3%), 3 different serotypes in 5/30 (16.7%) and 4 different serotypes in 1/30 (3.3%). The mean number of serotypes found in each nasopharyngeal swab was 1.2 (range 1–2) for MS-PCR and 1.9 (range 1–4) for Realtime-PCR (p = 0.001; 95% CL = 0.33–1.11). Multiple serotypes were never found in blood or CSF or pleural fluid.

## Discussion

The present study indicates that Realtime-PCR can be used directly on clinical samples for *Streptococcus pneumoniae* serotyping and has a greater sensitivity than Multiplex sequential PCR in identifying and serotyping *Streptococcus pneumoniae* in any body fluid (blood, cerebrospinal fluid, pleural fluid, nasopharyngeal swabs).

At present, what is known about pneumococcal serotype distribution in invasive infections or carriage, is mostly based on cultural isolate typing [19]. Pneumococcal serogroup and serotype identification is performed by using large panels of expensive antisera by capsular swelling reaction [5], [6], [8]. However cross-reactions between serotypes can occur and some strains are difficult to serotype or even remain non-serotypable [7]–[9]. Molecular serotyping of isolates [20] adds specificity to the test but cannot be performed on culture-negative samples. Actually, it is well known that culture may often give false-negative results because of small sample volume, previous antibiotic therapy [10], or unsatisfactory conditions of sample transport and storage which can impair the viability of pathogens. In all the cases where culture growth is not obtained, also serotyping cannot be performed neither with Quellung reaction nor with molecular methods applied on isolates.

We previously demonstrated, in accord with Corless et al. [13] that Realtime PCR is more sensitive than culture or end-point PCR for diagnosis of pneumococcal infection [14]. As for serotyping we previously demonstrated that MS-PCR can be used directly on whole blood samples for *Streptococcus pneumoniae* serotyping so giving results where cultural methods fail [14]. Similar results have been obtained on cerebrospinal fluid, on culture-negative meningitis in children [15].

However, even though more sensitive than cultural methods, MS-PCR leaves a not-negligible percentage of cases as non-typeable. The comparison between the present data and data obtained in previous researches [14], [15] demonstrates that the percentage of cases which remained non-typeable with MS-PCR is low or null when cerebrospinal fluids or pleural fluids are used, but it significantly increases if other biological samples, such as blood or nasopharyngeal swabs are used (fig. 1).

Among molecular methods Realtime-PCR has probably the best performance in terms of sensitivity, specificity and rapidity [16], so that it has being used for more than 10 years over a large spectrum of fields in biological research [17], [21], [22]. The present study demonstrates that no difference in sensitivity exists between the two tests when used on isolates. On the contrary, when used on clinical samples from culture-negative invasive pneumococcal infection, Realtime-PCR appears significantly more sensitive than MS-PCR irrespective of the sample used: multiplex sequential PCR allowed pneumococcal serotyping in about two/third samples while Realtime-PCR allowed pneumococcal serotyping in over 90% samples using a panel of 21 primer/probe sets, with good concordance between the two tests. The lower sensitivity of MS-PCR compared with Realtime-PCR is particularly evident in samples with low bacterial load, as often happens in whole blood of patients with pneumonia.

As for nasopharyngeal swabs, both Realtime-PCR and MS-PCR evidenced the presence of multiple serogroups/serotypes of *Streptococcus pneumoniae*. However Realtime-PCR appears significantly more sensitive than MS-PCR. The data have been obtained in a population of severely ill children in whom an invasive bacterial infection was suspected. Therefore the presence of multiple serotypes/serogroups may not reflect the situation of healthy children and cannot be considered as a co-colonization.

The evaluation of multiple colonization has important implications in the study of pneumococcal carriage and serotype replacement. At present in carriage studies the single colony method is commonly used to determine the serotype by the Quellung reaction. When colonies of multiple morphologies are present, selection by morphology adds some information, but nevertheless both methods underestimate the true rate of multiple carriage [14], [15], [23]–[25]. Actually, based on the single-colony cultural method, a single serotype per patient can be found and multiple colonization, which is a frequent condition cannot be revealed [24], [25]. Colonization studies in which several colonies are studied give more complete information but are limited by the high cost. In order to draw conclusions on limited phenomena as initial serotype replacement, individuating multiple colonization will be mandatory in future studies and molecular methods could be an important tool.

The MS-PCR method used in the present study, using 31 primer couples, was able to identify 31 serogroups/serotypes. The panel for Realtime PCR included primers/probes for 21 serogroup/serotype. The difference is in favour of MS-PCR sensitivity (and therefore it obviously goes toward the null hypothesis of the study which was aimed to demonstrate the higher sensitivity of Realtime PCR in serotyping directly from clinical samples) and nonetheless the higher sensitivity of Realtime PCR has been demonstrated. Actually, the 10 serogroup/serotypes not included in the Realtime PCR panel were expected to be responsible, according to pneumococcal epidemiology of European countries of less than 10% cases.

Potentially other primer/probe sets could have been used in the present study and a further decrease in the rate of non-typeable pneumococcal infection could be achieved. However even though Realtime-PCR and MS-PCR are undoubtedly less expensive than serological methods (respectively 1∶1.4 and 1∶2.3 for Realtime-PCR or MS-PCR vs serologic methods for 21 serotypes) (data not shown), the cost of the assay can be kept down accepting a small percentage of non-typeable samples similarly to what routinely occurs with serological methods. Actually, seen the epidemiological distribution of pneumococcal serotypes in Europe and USA [26]–[28], a further increase in the rate of typeable samples of only 5% would probably have required a disproportionate effort.

RT-PCR is a very sensitive methods and can help in diagnosis and serotyping of IPD. However, when a positive result is obtained in samples considered negative by the established ‘gold standard’ (culture), it might be speculated that this is due to poor specificity of PCR rather than improved sensitivity. Actually, in the past years, PCR was found positive in blood samples from healthy controls [29], [30]. However, the new techniques used in the present paper (Realtime PCR instead of end-point PCR) and the use of different target genes (lytA instead of ply) seem to eliminate the problem of false positive results described in the past. Moreover, in the present study, the diagnosis of pneumococcal infection done with the mosts specific [18] *lytA* gene was confirmed in MS-PCR by amplification of *cps* gene. Moreover, more than 90% of the samples could also be serotyped by Realtime PCR, using specific primers for each serotype, different from those for *cps* and *lytA*. Positive results from all these different targets in the same sample enhance confidence in the sample positivity.

The flexibility of the Realtime-PCR is useful to make the method suitable in clinical settings in different geographical areas [31]; actually primer/probe panel can be modified including primer/probe specific for serotypes which are present in a geographical area. Serotype 2, which has a negligible frequency in western countries, is prevalent in south-east Asia. As a consequence, primer/probes for serotype 2 could be designed and included in a Realtime panel more suitable for Asian countries [32]. Moreover distribution of pneumococcal serotypes may change over years, both as a consequence of immune pressure [33] and of secular trend [34], [35]. Therefore when the percentage of non-typeable samples increases new primer/probe sets targeting different serotypes can be added or substituted to the routinely used sets so maintaining a limited panel with affordable costs. This is important in any country and particularly useful in low-income countries, in which culture-based serotyping is barely affordable for the high costs and culture-negative cases have the highest rates [15].

The RT-PCR method for detecting bacterial infections due to *Streptococcus pneumoniae*, is simple, rapid, and more sensitive then previously used methods. The use of *lytA* gene (known to be extremely sensitive and specific) [18] in RT-PCR and *CpsA* gene in MS-PCR enhanced, in the study, confidence in sample positivity.

Other methods have been proposed for pneumococcal typing by molecular methods [36] but the presence of degenerate primers may reduced test sensitivity and the results may be difficult to interpret. As far as we could evaluate from the literature, the method presented in our study describes the largest panel of specific primer/probe sets for pneumococcal serotyping by Realtime-PCR and does not include any degenerate primer so to increase test specificity. Adjunctive tests can be useful to differentiate closely related serotypes [37] and serologic serotyping remains a useful tool [38].

In conclusion, both MS-PCR and Realtime-PCR appears have be able to obtain pneumococcal typing from isolates. Realtime-PCR is significantly more sensitive than MS-PCR in pneumococcal serogrouping/serotyping from clinical samples obtained from culture-negative invasive infections and in individuating presence of multiple pneumococci in nasopharyngeal swabs.

Distinction between different serotypes within the same serogroup may be necessary when, as in the case of 19F and 19A no cross-protection is induced between the two serotypes [39]. In other cases serotyping may be less important because vaccine-induced cross-protection between different serotypes within the same serogroup is known [39]. For this reason in the present work different specific primers for 19A or 19F have been included while serotypes 6A and 6B or serotypes within the group 18 were not distincted even though molecular techniques are available in our laboratory (data not shown). Based on those consideration a limited panel of 21 primer/probe sets has been designed with the aim to maintain an effective balance between expenses and epidemiological information. Actually, using a panel of 21 primers/probes, Realtime-PCR allows to achieve, today, pneumococcal serogrouping/serotyping in over 90% cases of culture-negative invasive pneumococcal disease in Italy and in most western countries where the serotype epidemiology is similar.

## Methods

Written informed consent was obtained from all parents or guardians; the study has been approved by the institutional review board of the Department of Pediatrics, Anna Meyer Children's Hospital and University of Florence, ITALY.

### Bacterial DNA Extraction

Bacterial genomic DNA was extracted from bacterial strains or biological samples using QIAmp DNeasy Blood & Tissue kit (Qiagen, Hilden, Germany) according to the manufacturer's instructions for extraction of a 200 µL sample.

### Molecular Diagnosis of *Streptococcus pneumoniae* Infection

The presence of *Streptococcus pneumoniae* DNA in biological samples and isolates was evaluated by molecular methods as previously described [14]. Primers and probes within the previously published autolysin gene *lytA* [14], [18] were used for RT amplification at a final concentration of 400 nM for primers, and 400 nM for JOE-labeled probes. The cycle threshold (C*_T_*) value is the PCR cycle number (out of 45) at which the measured fluorescent signal exceeds a calculated background threshold identifying amplification of the target sequence. If no increase in fluorescent signal is observed after 45 cycles, the sample is assumed to be negative. The presence of *Streptococcus pneumoniae* DNA was confirmed by the amplification of *CpsA* gene in multiplex sequential PCR as previously reported [10], [14] and briefly described in the next paragraph. Only samples positive for both *lytA* gene in RT-PCR and *CpsA* in MS-PCR were included in serotyping analysis.

### *Streptococcus pneumoniae* Isolates

Eight bacterial strains of *Streptococcus pneumoniae* were obtained from ATCC (respectively ATCC 6301 for 1, ATCC 6304 for 4, ATCC 6305 for 5, ATCC 10357 for 19A, ATCC 6323 for 23F, ATCC 6326 for 6B, ATCC 10368 for 9V, ATCC BAA-659 for 6A); 38 well characterized *Streptococcus pneumoniae* isolates from twenty-one serotypes (1, 3, 4, 5, 6, 7F, 8, 9V,10, 12F, 14, 15B/C, 18C, 19A, 19F, 20, 22F, 23F,33, 35B, 38) which had been isolated from blood and/or CSF and/or throat swab samples of patients and stored in germ bank at the Microbiology Laboratory of the Anna Meyer Children's University Hospital were used for molecular serotyping.

### Biological Samples

Sixty-nine biological samples (27 blood samples, 4 cerebrospinal fluid (CSF) samples, 4 pleural fluids ands 34 pharyngeal swabs), found positive for *Streptococcus pneumoniae* by Realtime-PCR with primer/probes from the *lytA* gene, as previously described [14], and obtained from children with culture-negative invasive pneumococcal infection were included in serotyping analyses both by multiplex-sequential PCR (MS-PCR) and Realtime-PCR.

### Case Definition

A patient with culture-negative invasive pneumococcal infection was defined as a patient in whom clinical signs of bacterial infections were associated with Realtime-PCR positivity for *lytA* gene (14) and negativity of cultural tests.

### Serotyping of Isolates and Clinical Samples

To prove sensitivity of the tests, both multiplex sequential PCR and Realtime-PCR were preliminarly performed on isolates.Then both methods were used directly on clinical samples from patients with a clinical suspicion of invasive bacterial infection. All the samples which had resulted positive with Realtime-PCR for lytA gene were included in serotyping analysis.

#### PCR serotyping by Multiplex sequential PCR

Briefly, thirty-one primer couples [14], [20], [37], [38] were grouped into nine multiplex reactions. *CpsA* (pneumococcal capsular polysaccharide synthesis gene) primers were included in all the reaction mix as a confirmatory test [14], [20], [37]. Amplification was performed in a Perkin-Elmer GeneAmp PCR system 2720 (Applied Biosystems, Foster City, CA, USA) under the following conditions: 95°C for 15 minutes followed by 35 amplification cycles of 94°C for 30 seconds, 54°C for 90 seconds, and 72°C for 60 seconds. A final hold was performed at 72°C for 10 minutes. The PCR products were analyzed by gel electrophoresis on 2% NuSieve agarose gels (Cambrex Bio Science, Inc., Rockland, ME) in 1x TAE buffer. The sizes of the PCR products were determined by comparison with the molecular size standard (100-bp ladder; Novagen, Inc.).

Samples negative with both *CpsA* primers and primers specific for all serotypes were excluded from the study. If amplification of *CpsA* was present but no other PCR product could be determined, the sample was reported as non-typeable.

#### PCR serotyping by Realtime PCR

Primers and probes were designed using the ABI Primer Express Software Package based on previously published *CpsA gene* (Table 1). Primer/probe sets for Realtime PCR and MS-PCR were obtained from the same gene region. The primer/probe panel was chosen on the basis of pneumococcal epidemiology in European countries, with the aim to include serotypes/serogroups causing over 90% cases of invasive pneumococcal disease.

**Table 1:** Primer and probe sets for pneumococcal serotyping by Realtime PCR.

*Streptococcus pneumoniae* serotype	Forward primer	Reverse primer	Probe
1	Cgtgcggtaattgaagctatga	Tgtggccccagcaactct	JOE-tgcttgcccttgtatagggt
3	Ggtcagcagaaagtatgcattgg	Tcgtttatccagggtctgatga	JOE-tattggatgtggtttatcgtgaaga
4	Tgggatgacatttctacgcacta	Ccgtcgctgatgctttatca	FAM-tcctattggatggttagttggtga
5	Ttacgggagtatcttatgtctttaatgg	Cagcattccagtagcctaaaactaga	JOE-ttgtctcagcaactctatttggctgtggg
6A/B	Aagtttgcactagagtatgggaaggt	acattatgtccRtgtcttcgatacaag	FAM-tgttctgccctgagcaactgg
7A/F	Gatggcatgtggcaaacca	Tttgccctccttaatcatttcac	JOE-ttggctatcggcatggtggt
8	Ccactcatcagtttcccatatgttt	tcaataattgaagaagcgaacgtt	JOE-tgatggcagatgggttgggacgag
9V/A	Tggaatgggcaaagggtagta	Tcggttccccaagattttctc	FAM-ttaatcatgctaacggctcatcga
10 A/B	cctctcctatcaactattactcattatactacct	aataaccataagtccctagatcattcaaag	FAM-tcattacaactccctatgtgacacgggtctttt
12 A/B/F	Gattattcgcttgcctcttcatg	atagccgaaataagctttccagaa	FAM-atttgtaagcggaccgtgcgatt
14	Cgactgaaatgtcactaggagaagat	aatacagtccatcaattactgcaatactc	FAM-tgtcattcgtttgccaatacttgatggtctc
15	Ttgaatcaggtagattgatttctgcta	ctctaggaatcaaatactgagtcctaatga	FAM- ctccggcttttgtcttctctgt
18B/C	Cctgttgttattcacgccttacg	ttgcacttctcgaatagccttactc	FAM-aaccgttggcccttgtggtgga
19 A	Ttcgacgacgtatcagcttca	tcattgagagccttaacctcttca	JOE-acccaaaacggttgacgcattatact
19 B/F	Ggtcatgcgagatacgacagaa	tcctcatcagtcccaaccaatt	FAM-acctgaaggagtagctgctggaacgttg
20	aaagatactggctgaggagctatctatt	agtcaaaagtactcaaccattctgatatattc	FAM-aggataaggtctactttgtgggagttc
22 A/F	Tctctgaaatggttgttgaaggaa	tcgcatccgatagttcttgtga	FAM-caatggcttactggcaatcccaggaca
23F	Tgctatttgcgatcctgttcat	agagcctccgttgtttcgtaaa	FAM-tttctccggcatcaaacgttaag
33 A/F	Cgagagagaatatgagggaattgtta	Tctcaatccccgcatttactg	FAM-aggaaaactgtggtcacggttcg
35B	Gcatggaggtggagcataca	tgtaaagactgcacaactcgatataaaa	FAM-caatttaaacaatattagtaaagcgcaggtcaagcaaa
38	Gtcttacgtagaacctctctggatga	tggtcctacaagcgacatgtg	FAM-ttgccacagatttggaatattttggtcgg

(Primers included in the MS-PCR analysis were the following: 1, 3, 4, 5, 6A/B, 7F/A, 7C/B, 8, 9V/A, 10A, 11A/D/F, 12A/B/F, 14, 15A, 15B/C, 16F, 17F, 18A/B/C/F, 19A, 19F, 20, 22A/F, 23F, 31, 33A/F, 34, 35B, 35F, 38F [14], [20], [37], [38].

The RT amplification was performed in 25 µL reaction volumes containing 2x TaqMan Universal Master Mix (Applied Biosystem, Foster City, CA, USA); primers and JOE labeled probes were used at a concentration of 400 nM; FAM labeled probes at a concentration of 200 nM. Six µl of DNA extract was used for each reaction. All reactions were performed in triplicate. A negative control (both blood samples from healthy controls and sterile water samples) and a positive control (blood samples or cerebrospinal fluid samples known to be positive for Streptococcus pneumoniae both by cultural and molecular methods) were included in every run. DNA was amplified in an ABI 7500 sequence detection system (Applied Biosystem, Foster City, CA, USA) using, for all the primers couples, the same cycling parameters as follows: 50° for 2 min for UNG digestion, 95°C for 10 min followed by 45 cycles of a two-stage temperature profile of 95°C for 15 sec and 60°C for 1 min.

If no increase in fluorescent signal was observed after 45 cycles for any of the primer/probe set, the sample was assumed to be negative with the serotype specific primers and was reported as non-typeable.

### Statistical Analysis

Data were evaluated to determine how many samples were positive by each test (MS-PCR or Realtime PCR), as well as those positive by two tests and those positive by only one test. Agreement between the two tests was assessed by Cohen's kappa statistic, with values of 0.00 to 0.20 indicating poor agreement, 0.21 to 0.40 indicating fair agreement, 0.41 to 0.60 indicating moderate agreement, 0.61 to 0.80 indicating good agreement, and 0.81 to 1.00 indicating excellent agreement. Marginal homogeneity of the two tests was assessed by McNemar's test. Results were expressed as mean levels and standard deviations. All continuous variables were expressed as mean ± SD. Fisher exact test and Pearson's chi-square test were used when appropriate.

### External Quality Control

An External Quality Control for Real-time PCR and standard PCR is regularly carried out with excellent results in our Laboratory of Immunology where all the molecular test were performed.
